# Association between HIV and acquisition of rifamycin resistance with first-line TB treatment: a systematic review and meta-analysis

**DOI:** 10.1186/s12879-024-09514-7

**Published:** 2024-07-01

**Authors:** Nesbert Zinyakatira, Nathan Ford, Helen Cox

**Affiliations:** 1 Division of Medical Microbiology, Department of Pathology, Faculty of Health Sciences, University of Cape Town, Cape Town, South Africa; 2 Division of Public Health Medicine, School of Public Health, Faculty of Health Sciences, University of Cape Town, Cape Town, South Africa; 3 Health Intelligence, Western Cape Government, Department of Health, Cape Town, South Africa; 4 Centre for Infectious Disease Epidemiology and Research, Faculty of Health Sciences, University of Cape Town, Cape Town, South Africa; 5 Institute of Infectious Disease and Molecular Medicine and Wellcome Centre for Infectious Disease Research in Africa, University of Cape Town, Cape Town, South Africa

**Keywords:** Tuberculosis, Human immunodeficiency virus, Acquired rifamycin-resistance systematic review, Meta-analysis

## Abstract

**Background:**

Multi-drug or rifamycin-resistant tuberculosis (MDR/RR-TB) is an important public health concern, including in settings with high HIV prevalence. TB drug resistance can be directly transmitted or arise through resistance acquisition during first-line TB treatment. Limited evidence suggests that people living with HIV (PLHIV) might have an increased risk of acquired rifamycin-resistance (ARR).

**Methods:**

To assess HIV as a risk factor for ARR during first-line TB treatment, a systematic review and meta-analysis was conducted. ARR was defined as rifamycin-susceptibility at treatment start with rifamycin-resistance diagnosed during or at the end of treatment, or at recurrence. PubMed/MEDLINE, CINAHL, Cochrane Library, and Google Scholar databases were searched from inception to 23 May 2024 for articles in English; conference abstracts were also searched from 2004 to 2021. The Mantel-Haenszel random-effects model was used to estimate the pooled odds ratio of any association between HIV and ARR among individuals receiving first-line TB treatment.

**Results:**

Ten studies that included data collected between 1990 and 2014 were identified: five from the United States, two from South Africa and one each from Uganda, India and Moldova. A total of 97,564 individuals were included across all studies, with 13,359 (13.7%) PLHIV. Overall, 312 (0.32%) acquired rifamycin-resistance, among whom 115 (36.9%) were PLHIV. The weighted odds of ARR were 4.57 (95% CI, 2.01–10.42) times higher among PLHIV compared to HIV-negative individuals receiving first-line TB treatment.

**Conclusion:**

The available data, suggest that PLHIV have an increased ARR risk during first-line TB treatment. Further research is needed to clarify specific risk factors, including advanced HIV disease and TB disease severity. Given the introduction of shorter, 4-month rifamycin-based regimens, there is an urgent need for additional data on ARR, particularly for PLHIV.

**Systematic review registration:**

PROSPERO CRD42022327337.

**Supplementary Information:**

The online version contains supplementary material available at 10.1186/s12879-024-09514-7.

## Introduction

The World Health Organization estimates that 410,000 individuals develop multi-drug-resistant (resistance to both a rifamycin (commonly rifampicin) and isoniazid) or rifamycin-resistant tuberculosis (MDR/RR-TB) globally each year [1]. The MDR/RR-TB burden is likely driven by both directly transmitted and acquired resistance, with modelling studies suggesting that direct transmission is the predominant epidemic driver [2–4]. However, resistance acquisition continues to fuel the epidemic and may also be significant across many settings [5–7]. Strategies that both interrupt transmission and reduce resistance acquisition are therefore required to tackle the epidemic [4, 8].

Several risk factors have been described for acquired MDR/RR-TB, including the use of inadequate treatment regimens either due to undiagnosed and/or untreated pre-existing drug resistance, poor prescribing practices, interrupted or incomplete treatment and pharmacokinetic factors [4, 7, 9]. According to one systematic review, patients with any baseline or pre-treatment first-line TB drug resistance were almost five times more likely to develop MDR/RR-TB compared to those with baseline pan-susceptible profiles [7]. Intermittent versus daily treatment regimens have also been associated with higher rates of acquired MDR/RR-TB, especially when intermittent dosing is started during the intensive phase of treatment [10–12].

The rifamycins, – which include rifampicin, rifabutin and rifapentine, – are potent anti-tuberculosis drugs [13]. Rifampicin has been the more commonly used, while rifabutin has been incorporated into regimens for treating PLHIV to overcome drug-drug interactions with commonly used antiretroviral drugs [14–16]. New, 4-month TB treatment regimens that include rifapentine are now also recommended [17, 18]. While rifampicin has been included in the recommended standardised 6-month regimen for decades [18], rifabutin has been sometimes been used in intermittent TB regimens due to its longer half-life [16, 19].

Increased resistance acquisition, particularly to the rifamycins, has been described among people living with HIV (PLHIV) [10, 20–22]. These studies have primarily been small and describe increased acquired rifamycin-resistance (ARR), often associated with advanced HIV disease [8, 9, 21]. While some studies have showed no difference in ARR risk between rifabutin- and rifampicin-based regimens in PLHIV [10, 11], the use of intermittent regimens, particularly in the initial intensive treatment phase, was associated with increased ARR regardless of rifamycin used in one study [10], while low CD4 cell count and not the rifamycin used was associated with increased ARR among PLHIV in other studies [11, 19].

While HIV was shown to be associated with increased acquired drug resistance (ADR, any TB drug) during TB treatment in one systematic review [7], two other systematic reviews found no significant association between HIV and MDR-TB among individuals with previous TB treatment [23, 24], which is often assumed to reflect acquired resistance. These conflicting results have been attributed to small sample sizes, variations in TB and HIV burdens in the countries where the studies were conducted, and higher early mortality before resistance acquisition is detected among PLHIV. Advanced HIV disease, as indicated by a CD4 cell count below 200 cells/mm^3^, may lead to changes in gut permeability, resulting in reduced absorption of antituberculosis drugs [25, 26]. With the potential expansion of 4-month rifapentine-based TB treatment, describing ARR risk among PLHIV is crucial for optimizing treatment for the more than 671,000 PLHIV who are estimated to develop TB globally each year [1].

## Methods

We aimed to evaluate HIV as a risk factor for the acquisition of rifamycin-resistance during first-line, rifamycin-based TB treatment. The Preferred Reporting Items for Systematic Reviews and Meta-Analysis (PRISMA) guidelines were followed, and a completed PRISMA checklist has been included (Table S2). The review was registered in PROSPERO (crd.york.ac.uk/PROSPERO/ CRD42022327337).

### Search strategy

We searched the PubMed/MEDLINE, CINAHL, Cochrane Library, and Google Scholar electronic databases from inception to 23 May 2024, abstracts from conferences (International Union Against Tuberculosis and Lung Disease conferences, between 2004 and 2021) and reference lists of selected studies and other reviews to identify additional relevant studies. Our search was limited to studies published in the English language without any restriction on study setting. Medical Subject Headings (MeSH) terms (HIV and TB) and different combinations of text words combined by Boolean operators were used to search for the relevant studies in the databases. The search terms used included TB treatment, HIV, acquisition/acquire(d), incidence, emergence/development, risk factor, drug resistance, rifamycin/rifampicin/rifampin- resistant(nce) (RR-TB), multi-drug - resistant(nce) (MDR-TB), drug resistant(nce) (DR-TB), rifamycin/rifampicin/rifampin (Full detail in Appendix 1).

All the identified study references based on the search criteria were imported into Covidence [27] for title and abstract screening, full text review and data extraction (including risk of bias indicators). Two reviewers (NZ and HC) conducted title and abstract screening, followed by a full text review for potentially eligible articles. For studies where eligibility was ambiguous, inclusion was resolved through a consensus-based discussion.

### Inclusion criteria

We included observational cohort studies and randomised controlled trials (RCTs) that included individuals with bacteriologically confirmed TB receiving first-line TB treatment, where drug susceptibility testing (DST) was done for at least one rifamycin at treatment start and any time during treatment, at the end of treatment, or at TB recurrence within 12 months of post TB treatment.

We included studies which reported data where ARR, among individuals with rifamycin-susceptible TB at treatment start, could be described as an outcome by HIV status, even if this was not a specified aim of the study. Only studies that included both PLHIV and HIV-negative individuals and where the majority of individuals (more than 66%) had a known HIV status were taken forward for review.

### Data extraction and risk of bias assessment

Structured data extraction and risk of bias assessment forms were constructed in Covidence. Relevant data was extracted for all the studies that met the eligibility criteria. The Newcastle-Ottawa Scale (NOS) assessment tool [28] was used to assess the risk of bias for the observational studies; RCTs were also treated as observational studies for the purposes of this review (Appendix 2), and the risk of bias results are shown in Table S1. Two authors (NZ and HC) independently assessed the risk of bias. Extracted data included: study design, study period, study setting, description of the study population, TB DST conducted and timing, directly observed therapy provision, frequency of treatment provision (daily versus intermittent), baseline DR profile, treatment regimen, timing of ARR determination, ARR (stratified by HIV status), assessment of reinfection or superinfection as cause of ARR (where available), HIV status (including any reported effect measures), and general comments on confounding, exclusions, limitations and strengths. Authors from two more recent studies provided additional data on ARR among individuals with rifamycin and isoniazid susceptible TB at treatment start [29, 30].

### Statistical analysis

Review Manager 5.4.1 [31] was used for the meta-analysis. A random-effects model was used to analyse the extracted data to ensure that estimates were not overly influenced by heterogeneity between the studies. Using the Mantel-Haenszel random-effects model, the effect measure (odds ratio, OR) with 95% confidence intervals for each study was estimated. A pooled OR of ARR in a random effects meta-analysis irrespective of study design was estimated as the overall effect measure, stratified by HIV status of the study populations. The I^2^ statistic was used to measure the degree of heterogeneity between the studies. Funnel plots, Beggs’s and Egger’s tests were used to assess publication bias. Publication bias was assessed by examining the funnel plot of the odds ratios (OR) against the standard error (SE) of the logarithm of the OR for each study.

## Results

### Study selection

**Fig. 1:**
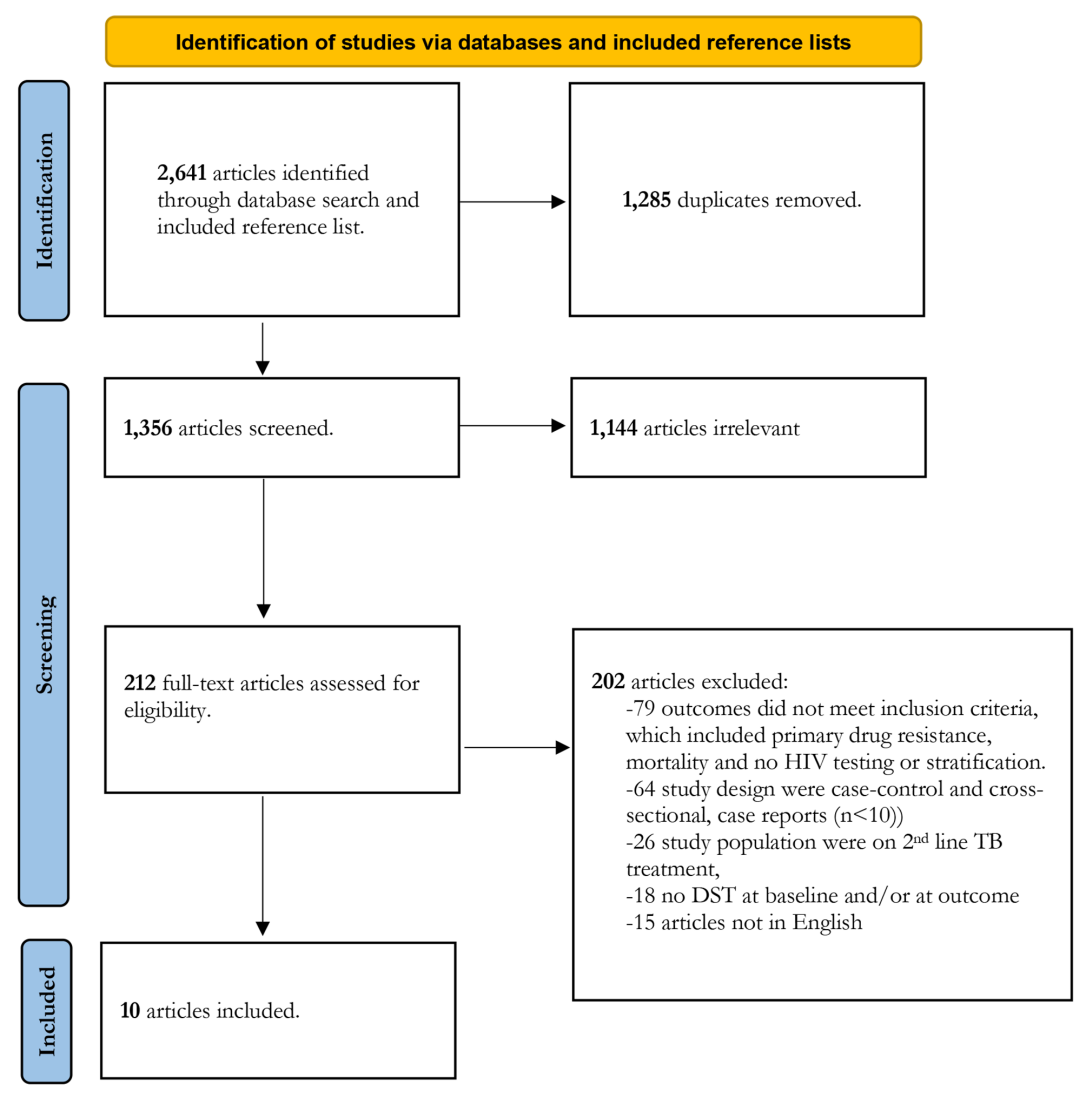
Study selection process flowchart

We identified a total of 2,641 articles through the electronic database search (35 of which were identified by screening the reference list of the included studies) (Fig. 1). Of these, 1,285 articles were excluded as duplicates. After title and abstract screening of the 1356 remaining articles, 1144 were excluded, leaving 212 for full text review (NZ and HC). Following full text review, 202 articles were excluded, leaving 10 eligible articles that met the inclusion criteria. The 10 eligible articles included one RCT (cross-protocol analysis), three prospective cohort studies and six retrospective cohort studies.

### Study characteristics

The ten included studies comprised five from the United States (US), two from South Africa, and one each from Uganda, India, and Moldova. Among these, six studies [10, 22, 30, 32–34] tested all individuals for HIV, while the remaining four had 11.8% [29], 17.3% [35], 20.7% [36] and 33.9% [11] individuals with unknown HIV status. Those with unknown HIV status, mainly from low HIV incidence settings, were grouped with HIV-negative individuals. In one study, individuals with unknown HIV status did not differ demographically and clinically compared to HIV-negative individuals [11], except for a higher median age. Another study, indicated that these individuals, although older, showed no disparities in TB outcomes and lacked specific risk factors for HIV [35]. However, the remaining two studies did not provide information on potential similarities or differences among the HIV groups [29, 36].

Table 1 shows characteristics of the included studies. In total, 97,564 individuals were enrolled across all studies, with 84,205 HIV-negative and 13,359 (13.7%) PLHIV; the proportion of individuals who were HIV-positive ranging from 4.7% [29] to 57% [30].

The included studies encompassed data collected between 1990 and 2014. Most of the combined data (94.4%) were contributed by studies from the US, while the two South African studies contributed 0.7%, and studies from Uganda, India and Moldova contributed 0.4%, 0.5% and 4.1%, respectively. After weighting according to sample size in each study, most of the combined data (49.8%) was contributed by US, while South Africa, Uganda, India and Moldova contributed 15.5%, 7.3%, 9.1% and 18.3%, respectively.

Only four studies reported adjusted and/or unadjusted effect measures (OR/ hazard ratios (HR)) of ARR by HIV status, primarily because ARR was not a primary study objective (Table 2). Three studies reported ARR separately among individuals with new and recurrent TB (defined at TB treatment start), while six studies did not differentiate ARR by either new or recurrent TB, and only one study focused specifically on new TB. Follow-up was generally until treatment completion or failure of treatment, with DST conducted at baseline and at the end of treatment. Some studies also conducted DST at multiple time points during treatment [22, 29, 33]. Additionally, four studies [10, 11, 30, 35] assessed ARR at TB recurrence, occurring anywhere from the end of treatment to 12 months post-treatment. In assessing reinfection as a cause of ARR, strains of TB (where available) were characterized through various genotyping techniques in five studies (Table 1) [11, 22, 30, 34, 35].

The standardized regimen for drug susceptible TB consisting of isoniazid, rifampicin (or rifabutin), pyrazinamide, and ethambutol in the intensive phase and isoniazid and rifampicin in the continuation phase [18] was used in all the studies; with small variations in treatment duration, use of rifabutin for some individuals instead of rifampicin in two studies [10, 11], and the inclusion of streptomycin in some regimens in two studies [29, 34]. Four studies provided daily treatment throughout, two studies did not specify treatment frequency, while the remainder had varying treatment frequency. The ten studies utilised directly observed therapy (DOT) with varied approaches and implementations (Table 1). In the intensive phase, five had continuous DOT, with three in the continuation phase. Additionally, three studies had variable DOT in the intensive phase, while most had either variable or partial DOT in the continuation phase. Treatment adherence was only assessed in two studies. One exclusively included individuals with greater than 80% adherence [22], and the other found no association between ARR and nonadherence [10].

### HIV status and risk of ARR

**Fig. 2:**
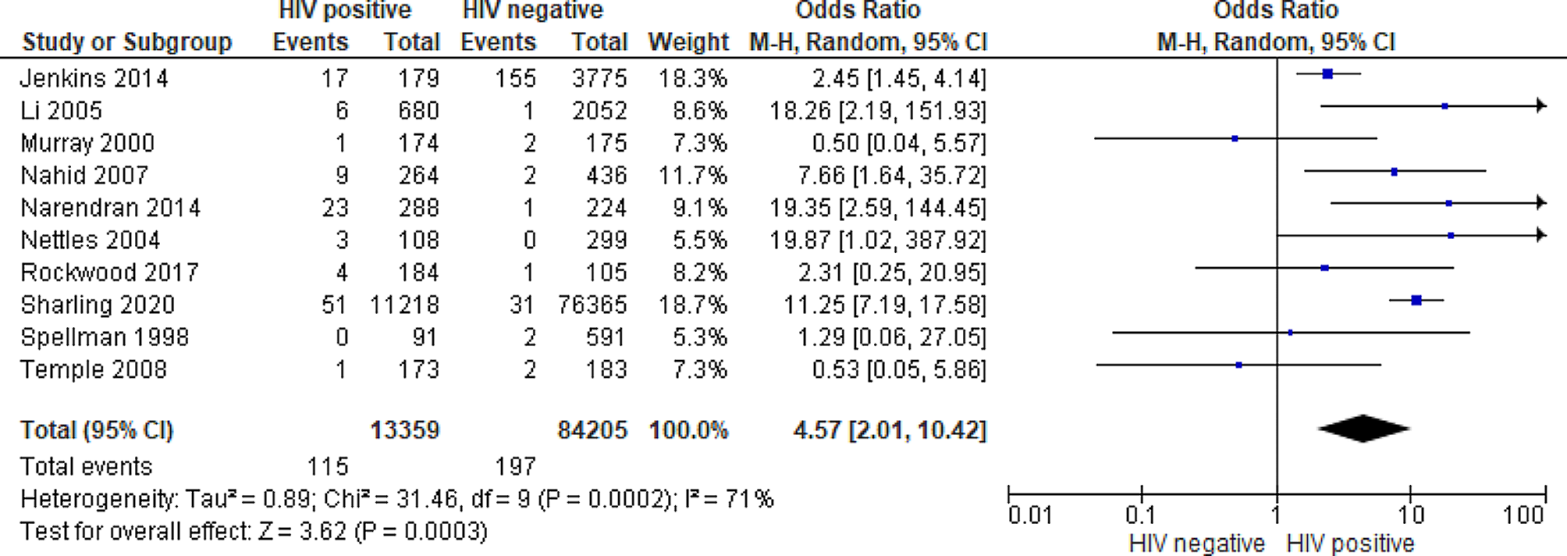
Forest plot of acquired rifamycin-resistance by HIV status (M-H random effects model)

HIV was identified as a risk factor for ARR in six [10, 11, 22, 29, 35, 36] of the ten studies, including the four studies [10, 22, 29, 36] that directly reported ARR risk by HIV. Rifamycin-resistance was acquired by 312 (0.32%) individuals during or after treatment, with 115 (36.9%) being PLHIV. Among PLHIV, 0.86% acquired rifamycin-resistance, compared to 0.23% among HIV-negative individuals. The crude risk of ARR was 3.70 (95% CI, 2.9–4.67) times higher in PLHIV than in HIV-negative individuals. The meta-analysis of the 10 studies showed HIV as a significant risk factor for ARR, with PLHIV 4.57 (95% CI, 2.01–10.42) times more likely to acquire rifamycin-resistance during or within 12 months of post first-line TB treatment compared to HIV-negative individuals (Fig. 2).

A sub-group analysis was conducted to explore differential risk, categorizing countries into high and low HIV prevalence settings. Among the three sub-Saharan African studies, one suggested higher ARR risk among PLHIV, while two found a lower ARR risk among PLHIV [30, 32, 34]. However, none were significant, and the pooled OR (0.90, 95% CI 0.23–3.47) was also not significant (Figure S1). Conversely, the seven studies from low HIV prevalence settings provided strong evidence that PLHIV have a higher risk of ARR compared to HIV-negative individuals, with a pooled OR of 7.37, (95% CI 3.03–17.92).

**Table 1:** Characteristics of included studies

Study	Study design	Setting and study period	New or recurrent patients and age range	Baseline drug resistance profile	Regimen	Daily versus intermittent treatment	Rifamycin	DOT	ARR determination	Assessment of reinfection as cause of ARR
Narendran [22]	Cross-protocol analysis of randomised controlled trial	India; 1999–2008	New, 15+	HRES susceptible	2HRZE_3_/4HR_3_	Thrice weekly	R	Throughout IP, partial in CP	Monthly during treatment and at treatment completion	MINU-VNTR, RFLP or spoligotyping available for 18/25 treatment failure; 14/18 identified as same TB strain.
Murray [32]	Prospective cohort study	South Africa; Jan 1995 - Dec 1995	Both, 18+	HRES susceptible	2HRZE/4HR	Daily	R	IP and CP	6 months post-treatment start	N/A
Nettles [11]	Prospective cohort study	US; Jan 1993 - Dec 2001	Both, 1-101	Rifamycin susceptible	2 weeks HRZE_7_ 6 weeks HREZ_2_ /HRfb_2_ or/HR_2_ (duration individualised) DOT over 50% in IP and CP	Daily for 2 weeks, twice weekly thereafter	R or Rfb	>=50% in IP and CP	2 months post treatment start, at treatment completion and at subsequent TB episode	RFLP available for 9/14 TB recurrence; all identified as same TB strain. All 3 ARR episodes were relapses
Rockwood [30]	Retrospective cohort study	South Africa; Mar 2013 - Jul 2014	Both, 18+	HR susceptible	2HRZE_7_ /4HR_7_ DOT in first 2 weeks only, SAT thereafter	Daily	R	First 2 weeks only	2, 5–6 months post treatment start and at subsequent TB episode (within 6 months post treatment start)	WGS available for 2/5 treatment failure or TB recurrences; 2 identified as reinfection.
Temple [34]	Prospective cohort study	Uganda; July 2003 - Nov 2006	Recurrent, 18+	R susceptible	2HRZES/1HRZE/5HRE	Daily	R	R	5, 8, 12 months post treatment start	RFLP used to confirm ARR in same TB strain; results not stated
Jenkins [29]	Retrospective cohort study	Moldova; Jan 2007 - Dec 2010	Both, 0+	HR susceptible	New: 2HRZE_7_/4HR Recurrent: 2HRZES_7_/1HRZE_7_/5HRE	Daily	R	IP and CP	2–3,3–4, 5 months post treatment start, and at treatment completion	N/A
Li [10]	Retrospective cohort study	US; Jan 1997-Dec 2000	Both, 0+	R susceptible or (E susceptible and H susceptible)	2HRZE or 2HRfbZE/4HR	Daily for 2 months, twice or thrice weekly thereafter	R, Rfb or R/Rfb	Variable in IP and CP	4 months post treatment start, at treatment completion and subsequent TB episode	N/A
Nahid [35]	Retrospective cohort study	US; Jan 1990-Dec 2001	Both, 18+	Pan-susceptible (Unknown drugs)	2HRZE_7_/4HR	Variable: daily, once, twice, thrice weekly	R, Rfb or Rpt	Variable in IP and CP	Treatment completion and subsequent TB episode	Genotyping available for 9/16 TB recurrences; all same TB strain. 2 had ARR and both HIV positive
Sharling [36]	Retrospective cohort study	US; 1998–2010	Both, 0+	HR susceptible	2HRZE/4HR	Note stated, likely variable	R	Variable in IP and CP	Treatment completion	N/A
Spellman [33]	Retrospective cohort study	US; Jan 1998-Dec 1995	Both, 0+	HRES, Rfb Cap Kan Eto susceptible	2HRZE/4HR	Not stated	Not stated	Throughout IP and CP	Periodically during treatment (timing not specified)	N/A

H = isoniazid, R = rifamycin, P or Rpt = Rifapentine, E = ethambutol, S = streptomycin, Z = pyrazinamide, IP = intensive phase, CP = continuation phase, DOT = directly observed therapy, SAT = self-administered therapy or unsupervised treatment, ARR = acquired rifamycin-resistance, ART = antiretroviral treatment, FDC = fixed dose combination, Rfb = rifabutin, WGS = whole genome sequencing, RFLP = restriction fragment length polymorphism

**Table 2:** Cohort numbers at baseline and ARR

Study	Susceptible to at least rifamycin at baseline					Isoniazid resistant and rifamycin susceptible at baseline: ARR/*N*			General comments
	HIV negative	HIV positive	ARR HIV negative	ARR HIV positive	Adjusted ARR risk for HIV positivity	HIV negative	HIV positive	Total (%)	
Narendran [22]	224	288 (56.3%)	1 (0.4%)	23 (8.0%)	aOR 2.02 (1.58–16.77)	0/15	0/26	0/41 (0.0%)	ART associated with substantially decreased ARR risk (higher than for HIV negative). Baseline low CD4 count, and baseline H resistance associated with ARR (OR: 13.3 (95% CI, 4.3–41.4))
Murray [32]	175	174 (49.9%)	2 (1.1%)	1 (0.6%)	N/A	3/	0/	3/71 (4.2%)	Study conducted among miners at high risk of reinfection (no distinction between relapse and reinfection)
Nettles [11]	299	108 (26.5%)	0 (0.0%)	3 (2.8%)	N/A	0/0	0/0	0/0 (0.0%)	Numbers excludes LTFU, death during treatment. HIV negative and HIV unknown groups combined. PLHIV on ART more likely to receive Rfb vs. R. Baseline low CD4 count, and baseline H resistance associated with ARR
Rockwood [30]	105	184 (63.7%)	1 (1.0%)	4 (2.2%)	N/A	0/10	0/7	0/17 (0.0%)	Additional data on HR susceptible patients at baseline obtained from authors. ART was associated with low risk of ARR. Advanced immunosuppression among 25% PLHIV; 32% of the men had prison history
Temple [34]	183	173 (48.6%)	2 (1.1%)	1 (0.6%)	N/A	2/	1/	3/29 (10.3%)	Only admitted patients in the study
Jenkins [29]	3775	179 (4.5%)	155 (4.1%)	17 (9.5%)	aHR 4.7 (1.2–17.8)	0/0	0/0	0/0 (0.0%)	Additional data on HR susceptible patients at baseline obtained from authors. Baseline H resistance associated with ARR (HR: 22.4 (95% CI, 8.1–61.8) in new patients; HR: 4.0 (95% CI, 1.4–11.9) in previously treated patients).
Li [10]	2052	680 (24.9%)	1 (0.0%)	6 (0.9%)	aOR 5.5 (1.4–21.5)	2/	1/70	3/70 (4.3%)	Use of a Rfb-based regimen and intermittent dosing associated with ARR. Low CD4 among PLHIV associated with ARR. Deaths during treatment excluded
Nahid [35]	436	264 (37.7%)	2 (0.5%)	9 (3.4%)	N/A	0/0	0/0	0/0 (0.0%)	Not clear whether ARR during treatment was assessed or just at relapse. HIV negative combined with HIV status unknown. PLHIV treated for significantly longer than HIV negative/unknown group.
Sharling [36]	76,365	11,218 (12.8%)	31 (0.04%)	51 (0.5%)	aRR 9.6 (6.9–13.3)	31/3 481	20/568	51/4 049 (1.3%)	HIV particularly associated with RMR-TB. ARR associated with non-US birth, prison residence and no DOT. Baseline H resistance associated with ARR (RR: 5.0 (95% CI, 2.8–8.7))
Spellman [33]	591	91 (13.3%)	2 (0.3%)	0 (0.0%)	N/A	1/23	0/1	1/24 (4.2%)	HIV common among drug users, middle aged adults, Blacks, born outside the US and people living in community-based facilities

H = isoniazid, R = rifamycin, Rfb = rifabutin, DOT = directly observed therapy, ARR = acquired rifamycin-resistance, ART = antiretroviral treatment, FDC = fixed dose combination, Rfb = rifabutin, LTFU = loss to follow-up

### Risk factors for ARR among PLHIV

Given the heterogeneity of included studies, small PLHIV cohort size, and relatively small ARR risk overall, assessment of risk factors for ARR among PLHIV was not possible. Nonetheless, two studies showed that ART provision significantly decreased the risk of ARR among PLHIV, although ARR risk remained higher than in HIV-negative individuals [22, 30]. Another study highlighted that PLHIV on ART were more likely to receive rifabutin instead of a rifamycin-regimen, but the risk of ARR was not described separately for the two regimens [11].

One study noted that the risk of ARR among PLHIV did not depend on the rifamycin (rifabutin versus rifampicin) used, but on the dosing schedule throughout the treatment period [10]. They found that PLHIV treated with rifampicin-based regimens alone had a higher risk of ARR if intermittent dosing of rifampicin was initiated during the intensive phase of treatment compared to PLHIV without intermittent dosing. This risk also remained in PLHIV with advanced HIV. In another study, an increased risk of ARR was also observed in PLHIV with intermittent treatment during the continuation phase, particularly among those with a low CD4 count [11]. While another study also found the incidence of ARR to be high among PLHIV with treatment failure and relapse with intermittent treatment [22].

Three studies identified baseline low CD4 cell count as a significant risk factor for ARR [10, 11, 22]. The low CD4 cell counts identified as risk factors in the studies were less than 100 cells/µl [10], a median of 51 cells/µl [11] and a median of 93 cells/µl [22]. There were three studies that reported ARR risk among individuals with pre-treatment rifamycin-susceptible and isoniazid-resistant TB, although this was not stratified by HIV status (Table 2) [22, 29, 36]. In all three studies, pre-treatment isoniazid-resistance was associated with significantly increased risk of ARR.

### Potential confounding of ARR risk among PLHIV

Given the potential for increased exposure to MDR/RR-TB during treatment among PLHIV, differentiating true resistance acquisition from superinfection with MDR/RR-TB is relevant. Among the five studies where genotyping was used to assess reinfection or superinfection with MDR/RR-TB, one study identified the same TB strain in all three ARR instances [11]. Another study, identified the same TB strains in 9 of 16 individuals with recurrent TB, including 2 ARR instances out of the total 11 in the study, both in PLHIV [35]. However, the other three studies did not report the genotyping results for any ARR instances [22, 30, 34], and therefore could not accurately identify true ARR.

Among the five studies from the US, HIV was proportionally more common among substance users, middle aged adults, African-American individuals, those born outside the US and people living in community-based facilities and prisons. Unfortunately, none of these studies reported whether these factors confounded any association between ARR and HIV. However, one study indicated that ARR was more common in prisoners, but they did not report by HIV-status [36]. Additionally, PLHIV were treated for significantly longer durations compared to the HIV-negative group in one study [35].

One of the three African studies included male migrant (internal and international) gold miners residing in hostels on mining sites, where there was a high prevalence of HIV-infection and particularly high TB transmission [32]. This study did not distinguish between TB reinfection and relapse. Individuals self-reported TB symptoms, and active case finding involved contact tracing of men who shared dormitories with individuals with diagnosed TB disease.

The second African study included previously treated TB individuals presenting for treatment at a hospital in Kampala, Uganda, a national referral center, with 30% referred from various parts of the country [34]. They were initially hospitalised, with admission recommended in the first two months of treatment, and later received DOT at primary healthcare facilities. These individuals potentially faced an elevated risk of nosocomially transmitted MDR/RR-TB [22], and the study population may not be representative of recurrent TB because of selection and referral biases [34]. Finally, three studies excluded individuals lost to follow-up and deaths during treatment in the denominator for ARR risk [10, 11, 29], while this is unclear in the other seven studies.

### Heterogeneity and publication bias

There was substantial statistical heterogeneity (I^2^ = 71%) using the random-effects model as expected for observational studies; nevertheless, the forest plot in Fig. 2 displays consistent point estimates across all but two studies [32, 34]. These two studies, both conducted in sub-Saharan Africa with a high HIV prevalence, found that HIV-negative individuals to be at a higher risk than PLHIV, although this was not significant in either study. In the subgroup analysis, there was no statistical heterogeneity for studies from Africa (I^2^ = 0%), while there was significant heterogeneity in studies from other regions (I^2^ = 74%) (Figure S1).

**Fig. 3:**
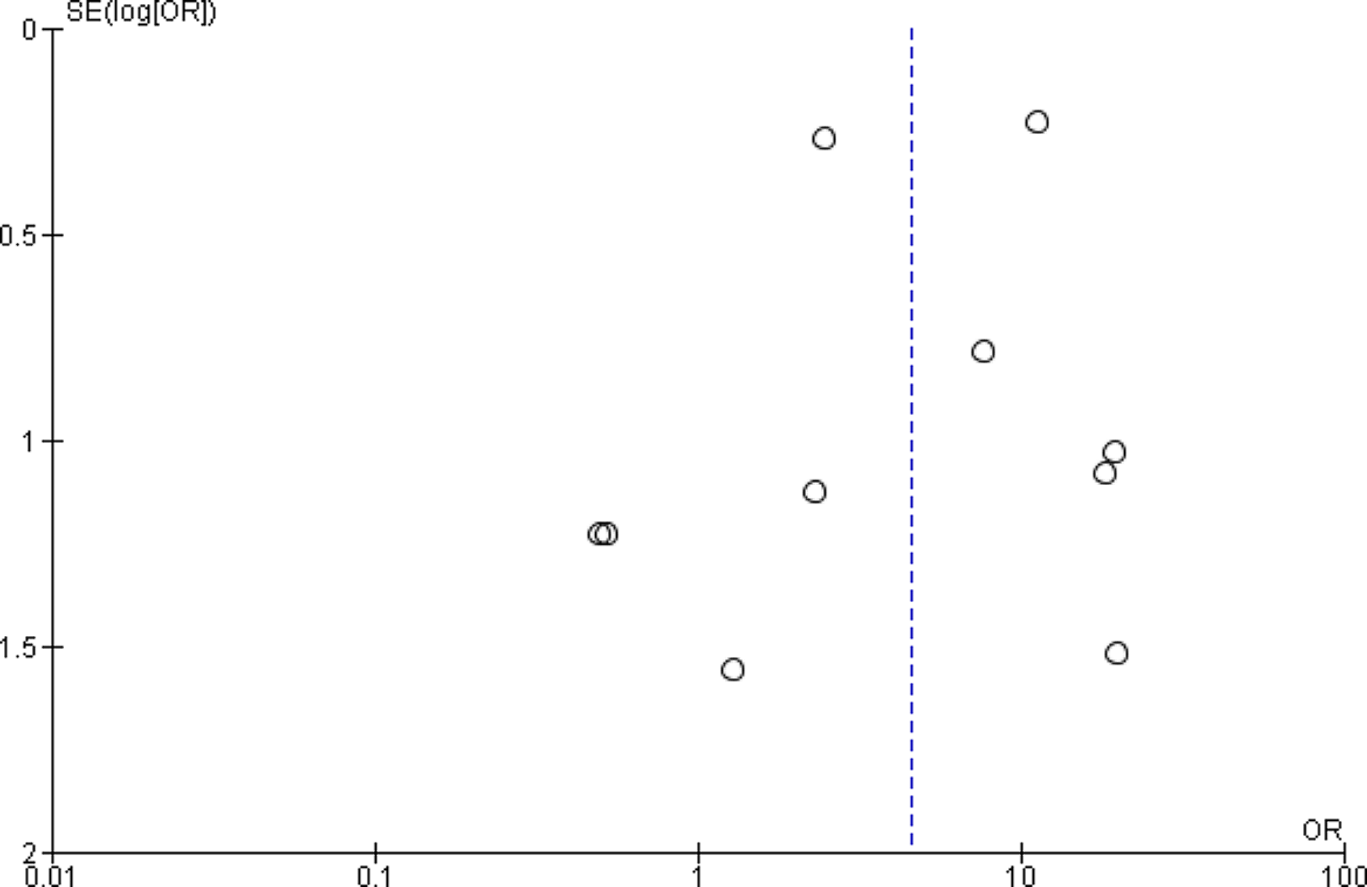
Funnel plot of the risk of acquired rifamycin-resistance studies included in meta-analysis

The funnel plot (Fig. 3) exhibits an asymmetrical distribution of the effect estimates, suggesting potential publication bias; however, both the Egger (*p* = 0.569) and Begg (*p* = 0.283) tests indicated no evidence of publication bias. The heterogeneity observed could be attributed to variability in study design, demographics, clinical characteristics, and methodological quality.

## Discussion

This systematic review suggests that PLHIV are over four times more likely than HIV-negative individuals to develop rifamycin-resistance while receiving rifamycin-based first-line TB treatment. While the overall proportion of PLHIV who acquired resistance was relatively low, in high TB burden settings where the majority of individuals with TB are living with HIV, this small proportion nevertheless represents a substantial absolute number of individuals and this may be sufficient to drive the continued emergence of MDR/RR-TB [3, 37, 38].

The majority of available data were obtained from low TB burden settings (notably the US) with only three smaller studies from the African region. Additionally, no data was available from after 2014 and most studies were not able to distinguish between true ARR or reinfection with rifamycin-resistant TB strain. Nonetheless, these findings align with a previous systematic review indicating a 3-fold increased risk of acquisition of any TB drug resistance among PLHIV [7]. Notably, the previous review focused primarily on the presence of pre-treatment drug resistance, along with HIV, as risk factors for acquired resistance to any TB drug and only included studies with at least one risk factor for resistance acquisition during treatment.

There were only three small studies from high HIV prevalence countries in Africa, and these showed inconsistent results. Two of the three African studies suggested that HIV-negative individuals might have a higher ARR than PLHIV. Importantly, these two studies had distinct populations: men working in South African gold mines and previously treated TB individuals hospitalized to receive treatment at a national referral and TB treatment hospital in Uganda. These populations are likely to be at a greater risk of reinfection with an already resistant MDR/RR-TB strain, which may be subsequently observed as acquired drug resistance. Neither study confirmed ARR using genotyping approaches.

The negative association in these two studies may also be attributed to a higher proportion of PLHIV who acquire resistance and subsequently die before ARR is detected [7], with 89 deaths per 1000 population [32] in one of the studies. Unfortunately, neither study specified if deaths were excluded from the denominator for ARR risk.

The US, with a relatively low TB and HIV prevalence [1], contributed around half of the available data, while the three African studies contributed less than a quarter of the overall data. In low HIV prevalence settings, such as the US, HIV primarily affects distinct populations, including men who have sex with men, people who inject drugs and ethnic minorities [39]. In contrast, in sub-Saharan Africa, HIV predominantly affects young people and women, reflecting a different population profile in mostly resource-limited settings [40]. Consequently, the overall weighted risk may not represent the majority of the global burden of TB among PLHIV.

All ten included studies were conducted between 1990 and 2014, a period when access to ART in resource-limited settings was limited (triple-drug ART was only available in 1996 in limited settings). Additionally, four of the five US studies were conducted in the late 1990s and early 2000s, when ART provision was less comprehensive [39]. Overall, insufficient data were reported on HIV treatment status, making it difficult to discern any effect of ART on ARR. Nonetheless, two studies reported a significantly lower ARR risk with ART provision [22, 30], with one concluding that despite the risk reduction, ART did not completely eliminate the risk of ARR [22].

The NOS assessment tool, primarily designed for nonrandomized studies, may not be appropriate for the single RCT we treated as an observational study. The NOS does not evaluate key aspects of randomization, assess blinding, or focus on biases such as confounding and selective reporting of outcomes [28, 41, 42]. Additionally, the scale used treats all criteria equally and overlooks methodological issues and rigor specific to RCT’s. The NOS is also subjective and open to interpretation, potentially leading to inconsistencies and variability in assessments between reviewers [42]. Nevertheless, as the trial was not designed to assess our research question (HIV as a risk factor for the acquisition of rifamycin-resistance) we considered it appropriate to treat this study as an observational study [43].

Genotyping of TB strains, both pre-treatment and at ARR occurrences, is necessary to accurately differentiate true ARR from reinfection [44]. Only five included studies reported any genotyping, with very limited data specifically for the ARR instances described. Nosocomial transmission of MDR/RR-TB may be an important cause of apparent emergence of resistance during treatment, particularly in countries, where all TB patients are hospitalized to receive treatment [29, 45]. In the study from Moldova, a country of low HIV prevalence and high MDR/RR-TB [1], PLHIV may have been particularly vulnerable to superinfection with MDR/RR-TB [46], although genotyping was not available [29].

In addition to the limitations of available data described above, this review has several other limitations. In most of the studies, ARR was a secondary outcome rather than the main research focus. Consequently, most studies were not adequately powered to investigate the HIV-ARR association. This likely affected data quality, statistical power, introduced bias, and hindered the ability to draw definitive and generalizable conclusions about the HIV-ARR association. Additionally, potential selection bias might have arisen from suboptimal sampling methods or inclusion criteria not ideal for assessing the impact of HIV on ARR risk. Moreover, confounders related to ARR risk, such as treatment adherence, PLHIV dying before ARR detection, ART status and duration, CD4 cell count, and co-morbidities were not adequately reported and therefore not able to be analysed. Additionally, while baseline isoniazid mono-resistance is a known risk factor for ARR [20, 47], insufficient data were available to assess the impact of isoniazid mono-resistance in the context of HIV.

Suboptimal adherence to TB treatment has been listed as a potential risk factor for acquired TB drug resistance generally, including PLHIV [7, 48]. However, there is limited data to support this assertion, with pharmacokinetic variation posited as a more likely cause [49, 50]. This proposition was supported by a meta-analysis published in 2012 and a subsequent study conducted in South Africa, both identifying pharmacokinetic variability as a risk factor for acquired drug resistance, particularly low rifampicin and isoniazid bioavailability even with perfect adherence [49, 51]. Further work describes the combination of pharmacokinetic variability and different TB lineages leading to varying propensities for acquiring resistance [52]. The extent to which varying pharmacokinetics contributes to ARR among PLHIV specifically remains unclear, with a systematic review unable to draw definitive conclusions as to the effect of HIV on first-line TB drug pharmacokinetics, due to the heterogeneity of available data [53]. Overall, the potential contribution of suboptimal adherence to ARR could not be assessed in this review. Firstly, there is no standardised method of measuring adherence, with different methods employed depending on the setting, disease burden, infrastructure and resources available [7]. While some included studies reported use of DOT versus self-administered therapy as a proxy measure for acceptable adherence, the use of DOT varied across included studies depending on previous TB treatment, treatment phase and dosing schedule. Significant heterogeneity was observed between the studies, especially from regions other than Africa, likely due to differences in study designs, population and clinical characteristics.

Despite the disproportionate burden of TB among PLHIV and the importance of the MDR/RR-TB in this vulnerable population, this review highlights the dearth of data on the emergence of resistance to rifamycins during TB treatment for PLHIV. While efforts to scale up to the 4-month rifapentine-based regimen and develop new shorter TB regimens are underway [17, 18, 54], efforts to characterize and mitigate the risk of ARR, both generally and among PLHIV, are urgently required. As it is unlikely that clinical trials will ever be sufficiently powered to assess resistance emergence as an outcome, analysis of large-scale routine observational data is likely to be informative, along with modelling approaches drawing on both clinical trial and observational data. Importantly, there is already substantial evidence that higher rifampicin doses are both safe and may lead to improved patient outcomes [55], and there are moves for a more individualised approach to TB treatment that takes into account variation in clinical characteristics such as TB disease severity and HIV status [56, 57]. These approaches should be pursued and are likely to provide more effective and more person-centred TB care.

### Electronic supplementary material

Below is the link to the electronic supplementary material.

Supplementary Material 1

## Data Availability

All data generated or analysed during this study are included in this published article and its supplementary information and can be obtained from the corresponding author at any time upon request.

## Abbreviations

ARR: Acquired rifamycin-resistant
ART: Antiretroviral treatment
CD4: Cluster of differentiation 4
CP: Continuation phase
DOT: Directly observed therapy
DST: Drug susceptibility testing
DR-TB: Drug resistant tuberculosis
E: Ethambutol
FDC: Fixed dose combination
H: Isoniazid
HIV: Human immunodeficiency virus
HR: Hazard ratio
IP: Intensive phase
LTFU: Loss to follow-up
MDR-TB: Multi-drug resistant tuberculosis
OR: Odds ratio
PRISMA: Preferred Reporting Items for Systematic Reviews and Meta-Analysis
PLHIV: People living with human immunodeficiency virus
NOS: Newcastle-Ottawa Scale
R: Rifamycin
RCT: Randomised controlled trial
Rfb: Rifabutin
RFLP: Restriction fragment length polymorphism
Rpt: Rifapentine
RR-TB: Rifamycin-resistant tuberculosis
SAT: Self-administered therapy
S: Streptomycin
SE: Standard error
TB: Tuberculosis
WGS: Whole genome sequencing
Z: Pyrazinamide
