# Supplementary material for: Association between HIV and acquisition of rifamycin resistance with first-line TB treatment: a systematic review and meta-analysis
Source: BMC Infect Dis. 2024 Jul 1;24:657. doi: 10.1186/s12879-024-09514-7 (PMC11218187; doi:10.1186/s12879-024-09514-7)
Supplement: Supplementary file 1 — Supplementary Material 1 [file 12879_2024_9514_MOESM1_ESM.docx]

**Supplementary materials**

**Appendix 1: Search strategy (search terms used)**

((((((((((((((((((((((((((((((((((((((((((((((((((((acquisition) OR (acquired)) OR (acquire)) AND (rifampicin)) OR (rifampicin resistant)) OR (rifampicin-resistant)) OR (rifampicin resistance)) OR (rifampicin-resistance)) OR (rifampin)) OR (rifampin resistant)) OR (rifampin-resistant)) OR (rifampin resistance)) OR (rifampin-resistance)) OR (acquired rifampicin resistant)) OR (acquired rifampicin-resistant)) OR (acquired rifampicin resistance)) OR (acquired rifampicin-resistance)) OR (acquired rifampin resistant)) OR (acquired rifampin-resistant)) OR (acquired rifampin resistance)) OR (acquired rifampin-resistance)) OR (drug resistant)) OR (drug resistance)) OR (multi drug resistant)) OR (multi-drug resistant)) OR (multi drug resistance)) OR (multi-drug resistance)) OR (multidrug resistance)) OR (multidrug resistant)) OR (MDR-TB)) OR (RR-TB)) OR (DR-TB)) AND (risk factor)) AND (incidence)) OR (incident)) AND (TB treatment)) OR (tuberculosis treatment)) AND (HIV)) OR (AIDS)) OR (HIV infection)) OR (HIV-1)) OR (HIV-2)) OR (hiv1)) OR (hiv2)) OR (human immunodeficiency virus)) OR (human immune deficiency virus)) OR (human immuno-deficiency virus)) OR (OR human immune-deficiency virus)) OR (acquired immunodeficiency syndrome)) OR (acquired immunodeficency syndrome)) OR (acquired immuno-deficiency syndrome)) OR (acquired immune-deficiency syndrome)) OR (sexually transmitted diseases)

**With mesh info**

((((((((((((((((((acquisition) OR (acquired)) OR (acquire)) OR (acquire)) AND (rifampicin)) OR (rifampicin resistant)) OR (rifampicin-resistant)) OR (rifampin)) OR (rifampin resistant)) OR (rifampin-resistant)) OR (drug resistant)) OR (multidrug resistant)) OR (multi-drug resistant)) OR (MDR-TB)) OR (DR-TB)) OR (RR-TB)) AND ( HIV Infections[MeSH] OR HIV[MeSH] OR hiv[tw] OR hiv-1*[tw] OR hiv-2*[tw] OR hiv1[tw] OR hiv2[tw] OR hiv infect*[tw] OR human immunodeficiency virus[tw] OR human immunedeficiency virus[tw] OR human immuno-deficiency virus[tw] OR human immune-deficiency virus[tw] OR ((human immun*) AND (deficiency virus[tw])) OR acquired immunodeficiency syndrome[tw] OR acquired immunedeficiency syndrome[tw] OR acquired immuno-deficiency syndrome[tw] OR acquired immune-deficiency syndrome[tw] OR ((acquired immun*) AND (deficiency syndrome[tw])) OR "sexually transmitted diseases, viral"[MESH:NoExp])) AND (risk factor)) AND ((TB treatment)OR(tuberculosis treatment))

**Appendix 2: Risk of bias**

Based on our research question, we developed a modified list of the Newcastle-Ottawa Scale risk of bias assessment tool, and for each item we defined

- Representativeness of the exposed cohort
- Selection of the non-exposed cohort- a star given if the HIV negative and HIV positive participants were selected from the same source population
- Ascertainment of exposure- a star given if DST conducted at beginning and at the end of first-line TB treatment (or in-between)
- Demonstration that outcome of interest was not present at the start of the study- a star give if no baseline drug resistance in study participants
- Comparability of cohorts on the basis of the design or analysis controlled for confounders – a star given if cohort available by HIV status, age and sex
- Assessment of outcome- a star given if outcome (ARR) was ascertained by DST
- Was follow-up long enough for outcomes to occur- a star given if follow-up period was at least 6 months (standard first-line TB treatment duration) or until event of interest (ARR) occurred
- Adequacy of follow-up of cohorts- a star given if at least 80% of all subjects accounted for

**Table S1: Risk of bias assessment using Newcastle-Ottawa for cohort studies**

|  |  |  |  |  | comparability | outcome | | |
| --- | --- | --- | --- | --- | --- | --- | --- | --- |
| **Reference (first author, publication year)** | Representativeness of the exposed cohort | Selection of the non-exposed cohort | Ascertainment of exposure | Demonstration that outcome of interest was not present at start of study | Comparability of cohorts on the basis of the design or analysis controlled for confounders | Assessment of outcome | Was follow-up long enough for outcomes to occur | Adequacy of follow-up of cohorts |
| Narendran 2014 ^22^ | * | * | * | * | - | * | * | * |
| Murray 2000 ^32^ | * | * | * | * | - | * | * | * |
| Nettles 2004 ^11^ | * | * | * | * | - | * | * | * |
| Temple 2008 ^34^ | * | * | * | * | - | * | * | * |
| Jenkins 2014 ^29^ | * | * | * | * | - | * | * | * |
| Li 2005 ^10^ | * | * | * | * | - | * | * | * |
| Nahid 2007 ^35^ | * | * | * | * | - | * | * | * |
| Rockwood 2017 ^30^ | * | * | * | * | - | * | * | * |
| Sharling 2020 ^36^ | * | * | * | * | - | * | * | * |
| Spellman 1998 ^33^ | * | * | * | * | - | * | * | * |

**Figure S1: Forest plot of acquired rifamycin-resistance by HIV status, subgroup analysis (M-H random effects model)**

**Table S2: Preferred Reporting Items for Systematic Reviews and Meta-Analyses (PRISMA) Checklist**

| **Section/ Topic** | **Item #** | **Checklist item** | **Reported on page #** |
| --- | --- | --- | --- |
| **TITLE** | | |  |
| Title | 1 | Identify the report as a literature review. | 1 |
| **ABSTRACT** | | |  |
| Abstract | 2 | Provide a structured summary including, as applicable: background; objectives; data sources; study eligibility criteria, participants, and interventions; study appraisal and synthesis methods; results; limitations; conclusions and implications of key findings.  See the [PRISMA 2020 for Abstracts checklist](http://www.prisma-statement.org/Extensions/Abstracts.aspx) for the complete list. | 1-3 |
| **INTRODUCTION** | | |  |
| Rationale | 3 | Describe the rationale for the review in the context of existing knowledge, i.e., what is already known about your topic. | 3-5 |
| Objectives | 4 | Provide an explicit statement of the objective(s) or question(s) the review addresses with reference to participants, interventions, comparisons, outcomes, and study design (PICOS). | 5 |
| **METHODS** | | |  |
| Eligibility criteria | 5 | Specify the inclusion and exclusion criteria for the review and how studies were grouped for the syntheses with study characteristics (e.g., PICOS, length of follow-up) and report characteristics (e.g., years considered, language, publication status) used as criteria for eligibility, giving rationale. | 6-7 |
| Information sources | 6 | Specify all databases, registers, websites, organisations, reference lists and other sources searched or consulted to identify studies. Specify the date when each source was last searched or consulted. | 5-6 |
| Search strategy | 7 | Present the full search strategies for all databases, registers and websites, including any filters and limits used. | 5-6, Appendix 1 |
| Selection process | 8 | State the process for selecting studies (i.e., screening, eligibility).  Specify the methods used to decide whether a study met the inclusion criteria of the review, including how many reviewers screened each record and each report retrieved, whether they worked independently, and if applicable, details of automation tools used in the process. | 6-7 |
| Study risk of bias assessment | 11 | Specify the methods used to assess risk of bias in the included studies, including details of the tool(s) used, how many reviewers assessed each study and whether they worked independently, and if applicable, details of automation tools used in the process. | 7-8, Appendix 2 |
| **RESULTS** | | |  |
| Study selection | 16a | Describe the results of the search and selection process, from the number of records identified in the search to the number of studies included in the review, ideally using a flow diagram. | 8-9,  Figure 1 |
| Study characteristics | 17 | Cite each included study and present its characteristics (e.g., study size, PICOS, follow-up period). | 10-12, Table 1 |
| Risk of bias in studies | 18 | Present assessments of risk of bias for each included study. | 19-22, Figure 3, Table S1  Figure S1 |
| Results of individual studies | 19 | For all outcomes, present, for each study: (a) summary statistics for each group (where appropriate) and (b) an effect estimate and its precision (e.g. confidence/credible interval), ideally using structured tables or plots. | 12-13, 22, Figure 2, Figure S2 |
| **DISCUSSION** | | |  |
| Discussion | 23a | Provide a general interpretation of the results in the context of other evidence. | 23-25 |
|  | 23b | Discuss any limitations of the evidence included in the review. | 23-27 |
|  | 23c | Discuss any limitations of the review processes used. | 23-27 |
|  | 23d | Discuss implications of the results for practice, policy, and future research. | 27-28 |
| **OTHER INFORMATION** | | |  |
| Registration and protocol | 24a | Provide registration information for the review, including register name and registration number, or state that the review was not registered. | 3, 5 |
|  | 24b | Indicate where the review protocol can be accessed, or state that a protocol was not prepared. | 5 |
|  | 24c | Describe and explain any amendments to information provided at registration or in the protocol. | N/A |
| Support | 25 | Describe sources of financial or non-financial support for the review, and the role of the funders or sponsors in the review. | 30 |
| Competing interests | 26 | Declare any competing interests of review authors. | 29 |
| Availability of data, code, and other materials | 27 | Report which of the following are publicly available and where they can be found: template data collection forms; data extracted from included studies; data used for all analyses; analytic code; any other materials used in the review. | 7-8, 28-29 |
